# Performance Evaluation of *Chlorococcum* sp. in Various Photobioreactor Designs: Impact on Biomass Production and Nutrient Removal

**DOI:** 10.3390/bioengineering13040388

**Published:** 2026-03-27

**Authors:** Rieza Zulrian Aldio, Nur Aqidah Donglah, Zubair Hashmi, Juliana Zaini, Muhammad Saifullah Abu Bakar, Muhammad Roil Bilad

**Affiliations:** 1 Faculty of Integrated Technologies, Universiti Brunei Darussalam, Jalan Tungku Link, Gadong BE1410, Brunei; 23h1311@ubd.edu.bn (R.Z.A.); 24h1302@ubd.edu.bn (N.A.D.); 22h8910@ubd.edu.bn (Z.H.); saifullah.bakar@ubd.edu.bn (M.S.A.B.); roil.bilad@ubd.edu.bn (M.R.B.); 2 Faculty of Engineering, Universitas Islam Riau, Jl. Kaharuddin Nst No.113, Pekanbaru 28284, Indonesia; 3 Faculty of Applied Science and Education, Universitas Pendidikan Mandalika, Jl. Pemuda No 59A, Mataram 83126, Indonesia

**Keywords:** *Chlorococcum* sp., bioremediation, biomass production, nutrient removal, photobioreactor

## Abstract

This study examines the influence of photobioreactor (PBR) configuration on the cultivation performance of *Chlorococcum* sp. using aquaculture wastewater as the growth medium. Four systems were compared: horizontal without aeration (H-Plain), horizontal with aeration (H-Aerated), vertical with aeration (V-Aerated), and vertical with aeration and red LED illumination (V-LED). Over 14 days, the V-LED system achieved the highest biomass concentration (0.50 g L^−1^) and volumetric productivity (0.063 g L^−1^ day^−1^), accompanied by nitrate and phosphate removals of 94% and 55.6%, respectively. Statistical analysis (ANOVA, *p* < 0.05) confirmed significant differences among configurations, demonstrating that light quality and aeration act synergistically to enhance growth and nutrient assimilation. While aeration improved CO_2_ transfer and mixing, it was insufficient without adequate photon delivery. Conversely, red LED illumination mitigated photolimitation in vertical systems, promoting efficient photosynthesis and nutrient uptake. Energy assessment revealed that V-LED offered the highest productivity in expense of power input (1.08 kWh day^−1^). These findings highlight the critical role of integrated PBR design, emphasizing that optimal combinations of geometry, aeration, and spectral lighting as keys to achieving high biomass yields and efficient nutrient removal in sustainable microalgae-based wastewater treatment systems.

## 1. Introduction

Species of the green microalgal genus *Chlorococcum* have been widely investigated for phycoremediation, with various strains reported to remove nitrogen, phosphorus and organic pollutants from municipal, industrial and aquaculture wastewaters while simultaneously generating biomass for biofuel or other bioproducts. This dual role makes *Chlorococcum* sp. attractive candidates for integrated wastewater treatment and resource recovery. Several strains also accumulate carotenoids with potential applications in food, cosmetic and pharmaceutical products. Furthermore, microalgal cultivation in photobioreactors (PBRs) offers a controlled environment that can enhance nutrient uptake, photosynthetic activity and biomass yield, thereby improving the overall efficiency of wastewater treatment systems [1,2,3,4,5].

Recent advancements in PBR technology have focused on optimizing several key parameters, including reactor design, light quality, and aeration intensity, to enhance microalgae growth and nutrient removal. Among these factors, reactor geometry, whether horizontal or vertical—plays a significant role in determining the effectiveness of light penetration, mass transfer, and nutrient assimilation [6,7]. Horizontal PBRs (HPBRs) typically provide better light distribution and nutrient mixing, which are essential for enhancing photosynthetic efficiency and algal growth compared to vertical configurations [8]. Despite numerous studies investigating phycoremediation and biomass production using different *Chlorococcum* species [1,2,4], comparative assessments that cultivate *Chlorococcum* sp. in aquaculture wastewater across multiple PBR configurations operated side by side remain relatively limited. This is critical for improving wastewater treatment systems and achieving higher biomass yields.

Several studies have focused on optimizing individual factors like light intensity, aeration, and nutrient concentration. For example, red LED lighting could enhance photosynthetic efficiency by aligning with the absorption peaks of chlorophyll, promoting increased biomass production in microalgae [9,10]. Aeration systems, on the other hand, improve gas transfer and nutrient distribution, which are essential for maximizing microalgal growth and nutrient uptake in PBRs [11]. However, it remains unclear how the integration of these factors across different PBR designs affects the growth of *Chlorococcum* sp., especially in terms of nutrient removal and biomass productivity. Therefore, a more holistic and comparative approach is required to evaluate the combined impact of reactor geometry, lighting systems, and aeration strategies on *Chlorococcum* sp. cultivation.

The main research problem centers on understanding how various PBR configurations and operational conditions affect the growth and nutrient removal efficiency of *Chlorococcum* sp. The microalgae growth in a PBR is affected by many factors, but the extent of those factors in affecting the growth is poorly reported in the literature. While previous studies have provided insights into the effects of individual parameters [12,13], there is a need for direct comparisons of these factors under similar conditions. Furthermore, it is crucial to evaluate how these parameters interact in different PBR designs to determine the optimal conditions for both biomass productivity and nutrient uptake. Addressing these challenges will enhance our understanding of how to design more efficient PBR systems for wastewater treatment and biomass production [14,15]. It is widely accepted that a specific parameter acts as the limiting factor that dictates the growth rate, but as later found in this work, all factors are interrelated, resulting in collective parameters that serve as the growth-limiting factor, dictating the growth rate. Still, all factors are interrelated, leading to collective parameters that limit growth.

Previous research has explored various methods to enhance algal growth, including optimizing light exposure with artificial lighting (e.g., LEDs) [16], improving aeration for better gas transfer, and adjusting nutrient concentrations resulting in enhanced growth rates [10,17]. Similarly, aeration improved nutrient availability and biomass productivity by facilitating gas exchange, thereby promoting microalgal growth [18,19]. In terms of reactor design, horizontal PBRs are more efficient in terms of light distribution and mass transfer compared to vertical PBRs, which may suffer from shading and reduced light penetration, particularly in dense cultures [6]. However, while these individual factors have been studied extensively, there is a need for comparative research that evaluates how they interact in the cultivation of *Chlorococcum* sp.

This study aims to compare four practically relevant PBR configurations—horizontal and vertical, with and without aeration and artificial lighting—for cultivating *Chlorococcum* sp. in aquaculture wastewater. Rather than implementing a fully balanced factorial design, it evaluates these representative operating systems to examine how geometry, aeration and light jointly influence biomass productivity and nutrient removal. The findings could offer practical design recommendations for optimizing PBR systems used in wastewater treatment and biomass production.

## 2. Materials and Methods

### 2.1. PBR Design and Experimental Setup

The experiment employed two types of PBRs—horizontal and vertical—to cultivate *Chlorococcum* sp. using non-sterilized aquaculture wastewater as the growth medium to simulate real aquaculture effluent. Four configurations were tested to assess their effects on algal growth and nutrient removal efficiency: (1) horizontal PBR (H-Plain) without aeration; (2) horizontally continuous aerated PBR (H-Aerated); (3) vertical PBR (V-LED) with aeration and red LED lighting; and (4) vertical PBR (V-Aerated) with aeration only. These configurations were chosen as feasible and representative operating modes under local constraints, so the comparison reflects typical horizontal and vertical systems rather than a strict geometry-only or fully factorial design.

The horizontal PBRs (measured 122 cm (length) × 40 cm (diameter) with a working depth of 16 cm) were constructed from stainless steel (SS302) and equipped with an openable lid for easy access (also SS302), with a total working volume of 58 L. Two operational modes were tested: a non-aerated system relying on natural light (H-Plain) and an aerated system using two aquarium air pumps (Venus Aqua AP-408A, power: 5W (Kancheepuram, Tamil Nadu, India) supplying air at total of 8 L min^−1^ (with a specific aeration rate of approximately 0.14 L L^−1^ min^−1^ for these PBRs) through spherical ceramic diffusers model D892/D1881 (Universal Analytical Technology Pte. Ltd., Singapore), producing fine bubbles (~3 mm diameter) under natural light (H-Aerated), with natural light condition measured averagely at 30 µmol photons m^−2^ s^−1^ (PBR lid is always opened). Using only two point diffusers across the 122 cm tank length is a practical limitation and may have resulted in non-uniform gas distribution; future designs could consider extended or flexible diffusers to improve aeration uniformity along the reactor.

The vertical PBRs (measured 15 cm diameter × 90 cm height), constructed from transparent acrylic for optimal light transmittance, had a working volume of approximately 13.8 L. They operated in two configurations: (1) continuous aeration only (V-Aerated) and (2) continuous aeration combined with supplemental artificial lighting from a 10 m red LED strip (Jiangmen Hongshe Lighting Technology Co., Ltd., Jiangmen City, China.) emitting 53 µmol photons m^−2^ s^−1^ (V-LED). The red LED strip was wrapped externally around the column along most of its height, providing lateral illumination of an upright cylindrical reactor. The addition of red LED lighting was intended to boost photosynthetic activity, especially in the vertically constrained design as tested earlier research [20]. As for the aeration, both were supplied by an air pump at 4 L min^−1,^ with a specific aeration rate of approximately 0.29 L L^−1^ min^−1^.

All systems were maintained in ambient environmental conditions, with temperatures ranging from 22 to 25 °C. The horizontal and vertical reactors (except V-LED) operated under natural daylight (12:12 h light/dark cycle), where irradiance fluctuated with time of day and weather. This variability was treated as part of realistic operation for open horizontal systems and was not compensated.

### 2.2. Cultivation Medium and Inoculation

The *Chlorococcum* sp. inoculum was obtained from laboratory-maintained cultures at Universiti Brunei Darussalam. A previously isolated *Chlorococcum* sp. strain was used, confirmed through light microscopy and comparison with published morphological descriptions; species-level placement has not yet been confirmed by molecular phylogenetic analysis, as reported earlier work [20]. Inoculation was carried out at a 1:25 volumetric ratio (inoculum: wastewater), corresponding to an initial cell density of 4280 cells mL^−1^ (~0.19 g L^−1^ dry biomass). Because non-sterile aquaculture wastewater was used as the growth medium, all reactors operated as algal–bacterial consortia rather than axenic microalgal cultures.

### 2.3. Monitoring and Analytical Procedures

Temperature, pH, dissolved oxygen (DO), and conductivity were monitored daily. DO was measured using a portable DO meter (DO-9100, Hanna Instruments, Woonsocket, RI, USA), and pH/conductivity were measured using a multiparameter instrument (HI5521, Hanna Instruments, Woonsocket, RI, USA).

**Figure 1:**
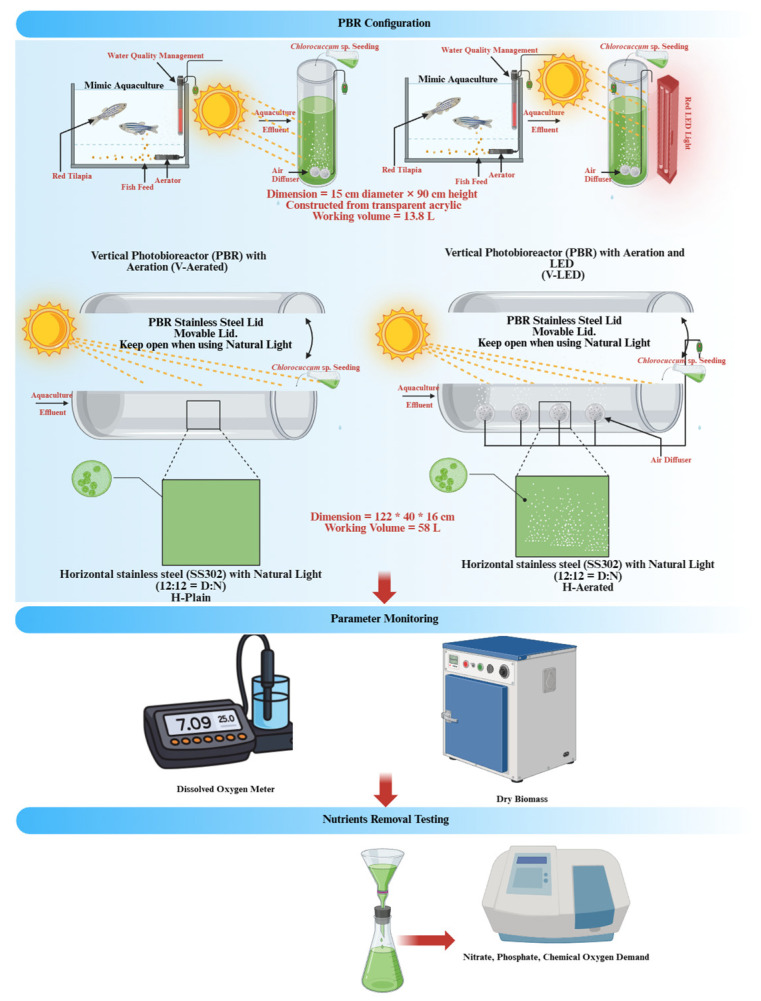
Overall processes and PBR (horizontal and vertical) setups used in the study.

Biomass was quantified by gravimetric dry weight measurements only, using Mettler Toledo analytical balance (model ME104, readability = ±0.0001 g using Shimadzu AT-R Series, Shimadzu Corporation Kyoto, Japan), where 5 mL triplicate samples filtered, rinsed with deionized water to remove salts, and dried at 105 °C until constant weight. Nutrients (Nitrate (NO_3_^−^), phosphate (PO_4_^3−^), chemical oxygen demand (COD)) were measured at start and end using commercial kits (HI93728-0, HI93713-0, HI93754A; Hanna Instruments, Woonsocket, RI, USA). Overall process of this study is shown in Figure 1.

### 2.4. Geometric and Productivity Calculations

To allow comparative analysis, several performance metrics were derived.

#### 2.4.1. Surface-to-Volume Ratio (S/V)

The surface-to-volume ratio (S/V) was calculated using Equation (1) [21]:

(1) $$S/V\;\mathrm{ratio}=\frac{A}{V}$$

where *A* is illuminated surface area (cm^2^) and *V* is working volume (cm^3^). For horizontal reactors, only the open liquid surface was included, while for vertical reactors the full lateral acrylic surface was considered illuminated.

#### 2.4.2. Volumetric Productivity

The volumetric productivity (Pᵥ) of biomass was determined using Equation (2) [22]:

(2) $$P_{v}=\frac{X_{\max}-X_{0}}{t}$$

where *X_max_* denotes peak biomass concentration (g L^−1^), *X*_0_ denotes initial biomass (g L^−1^), and *t* denotes days to peak.

#### 2.4.3. Specific Electrical Energy Consumption per Biomass

The specific electrical energy consumption per biomass (*E_b_*) was calculated using Equation 3 [23]:

(3) $$E_{b}=\frac{E_{t}}{P_{v}}$$

where *E_t_* is daily electrical energy input (kWh day^−1^) from aeration and LED lighting, and *P_v_* is daily biomass yield (g L^−1^ day^−1^). Incident solar radiation on the open horizontal reactors was not included, so the analysis reflects only operational electrical inputs rather than a full energy balance.

### 2.5. Statistical Analysis

All experiments were run in triplicate, and results are reported as mean ± standard deviation. Comparisons among systems were evaluated using one-way ANOVA with Tukey’s post hoc test (*p* < 0.05).

## 3. Results and Discussion

### 3.1. Biomass Growth Kinetics

**Figure 2:**
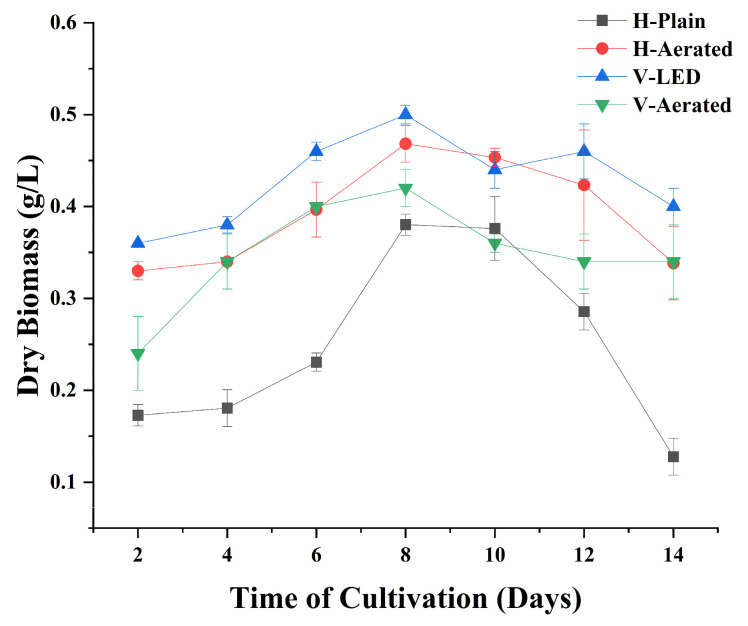
Biomass trajectories over 14 days cultivation period in four PBR configurations; H-Plain, H-Aerated, V-LED, and V-Aerated.

Dry biomass trajectories over the 14-day cultivation (Figure 2) showed that V-LED attained the highest biomass concentration (0.5022 g L^−1^, day 8), followed by H-Aerated (0.4683 g L^−1^, day 8), V-Aerated (0.4336 g L^−1^, day 8), and H-Plain (0.3803 g L^−1^, day 10). The results indicate that spectral management paired with better gas transfer effectively increased biomass accumulation than relying on geometry or aeration alone.

Statistical analysis confirmed significant differences among the four systems (ANOVA: F = 8.65, *p* = 0.00045). Tukey’s HSD post hoc test revealed that H-Plain differed significantly from all other configurations (*p* < 0.05), whereas V-LED, H-Aerated, and V-Aerated did not differ significantly from one another (Table S2 in Supplementary File). This outcome indicates that while V-LED achieved the numerically highest peak, its apparent advantage over H-Aerated was not statistically significant when replicate variability was considered.

Mechanistically, V-LED mitigated vertical photolimitation by supplying red photons aligned with chlorophyll absorption, improving optical utilization and stabilizing day-to-day growth despite longer light paths in the column [23,24,25,26]. H-Aerated reached the second-highest peak, consistent with improved CO_2_ supply and bulk mixing; however, aeration alone did not match V-LED, underscoring that mass-transfer gains must be coupled with adequate, spectrally appropriate light to yield sustained biomass increases. The V-Aerated plateau at 0.4336 g L^−1^ reflects persistent optical constraints in the lower column where added aeration could not compensate for photon scarcity. By contrast, H-Plain, despite a short optical path, displayed the lowest peak, consistent with carbon-supply and boundary-layer limitations in the absence of aeration that offset the geometric advantage. Reports on bubble-induced turbulence, shear, and CO_2_-regime shifts further explain why aeration requires tuning and co-optimization with light delivery to avoid diminishing returns [27,28,29]. Therefore, enhanced biomass production requires a synergistic interaction among reactor design, aeration, and lighting systems, rather than reliance on any single parameter.

Importantly, all systems operated under non-axenic conditions due to the use of raw aquaculture wastewater. Although bacterial populations were present, the steady rise in algal biomass (from an initial 0.1729 g L^−1^ to 0.3803–0.5022 g L^−1^) indicates that *Chlorococcum* sp. growth dominated the system. As microalgal biomass increased, competition for nutrients and oxygen supersaturation likely restricted bacterial proliferation. This competitive effect is consistent with prior studies showing that microalgae can suppress heterotrophic bacteria in mixed systems through rapid nutrient uptake and oxygen accumulation [30,31]. While bacterial activity may have contributed to COD removal, the observed biomass kinetics strongly support algal dominance as the primary driver of system performance.

Overall, the results demonstrate that light quality and aeration function synergistically rather than independently. These interpretations should be viewed with the understanding that biomass was assessed only by dry weight, as OD measurements were unavailable, which may introduce slight variability in the growth trends. Aeration enhances CO_2_ supply, but its benefits are only realized when adequate photons are delivered, whereas spectral enhancement alone is insufficient without proper mixing. These findings are consistent with previous reports where red light substantially improved biomass productivity and nutrient assimilation, supported by aeration effects [30,31,32,33].

### 3.2. Dissolved Oxygen

**Figure 3:**
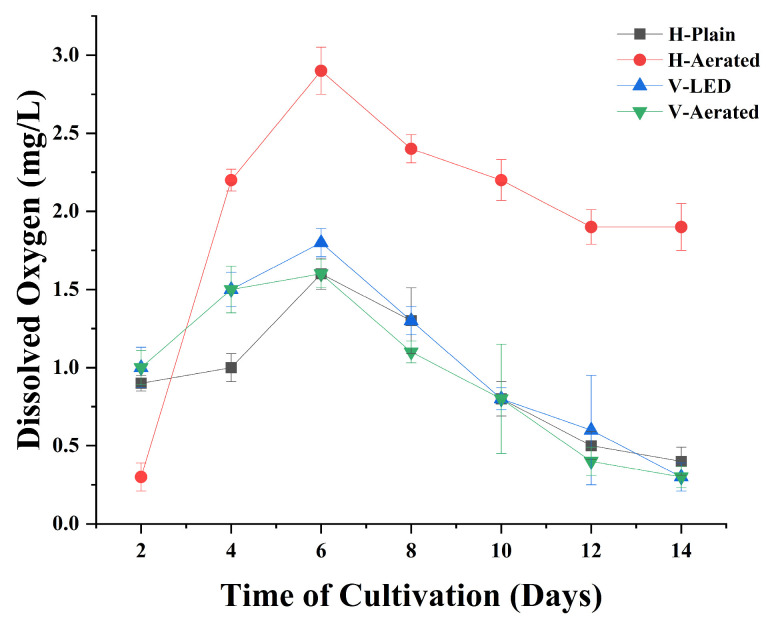
Evolution of dissolved oxygen of the microalgae broths from the four PBR configurations reflecting the growth over the cultivation time; H-Plain, H-Aerated, V-LED, and V-Aerated.

DO concentration significantly impacts microalgal metabolism, acting as both a byproduct of photosynthesis and a substrate for respiration. The DO values recorded in the four PBR configurations, shown in Figure 3, highlight the relationship between oxygen dynamics and *Chlorococcum* sp. growth.

The H-Aerated system achieved the highest DO concentrations, peaking at 2.9 mg L^−1^ on day 6 and maintaining levels above 1.9 mg L^−1^ until day 14. However, despite the high oxygen availability, biomass accumulation was moderate. This indicates that while aeration improved oxygen distribution, it may have caused mechanical stress from turbulence [34], limiting net photosynthetic efficiency [29,35]. In contrast, V-LED maintained DO levels between 1.0 and 1.8 mg L^−1^ during its active growth phase. These levels aligned with stable biomass increases, suggesting an effective balance between oxygen production from photosynthesis and its removal through aeration. The use of red LED lighting likely enhanced photosynthetic activity, contributing to consistent oxygen production and growth.

The H-Plain system, which operated without aeration, showed a steady decline in DO after peaking at 1.6 mg L^−1^ on day 6, decreasing to 0.4 mg L^−1^ by day 14. These drops aligned with the biomass peak on day 8, suggesting that while initial oxygen levels supported growth, limited mass transfer later caused oxygen depletion. This likely led to metabolic stress and reduced productivity. In contrast, the V-Aerated system maintained a relatively steady but lower DO level, never exceeding 1.6 mg L^−1^ and dropping to 0.3 mg L^−1^ by the end of cultivation. Despite aeration, the lack of artificial lighting appears to have hindered photosynthetic oxygen production [29], resulting in lower biomass yields and indicating an inadequate synergy between light input and gas transfer. These results confirm that aeration alone could not overcome photon scarcity in vertical columns, limiting both oxygen production and growth.

Importantly, the progressive increase in DO during early cultivation phases serves as a proxy for active photosynthetic reactions. Rising oxygen levels directly reflect algal activity and indirectly indicate that *Chlorococcum* sp. outcompeted bacterial populations for available nutrients. In non-axenic wastewater, bacteria typically consume oxygen through respiration, which would suppress DO if bacterial dominance were significant. Instead, the observed DO elevation confirms that photosynthetic oxygen release outweighed bacterial consumption, supporting algal dominance in driving system performance.

Overall, DO dynamics demonstrated that while aeration improved oxygen availability, it did not guarantee higher biomass unless paired with sufficient light. The most effective outcomes occurred in V-LED, where spectral enhancement provided a balanced oxygen regime aligned with robust algal growth.

### 3.3. Nutrient Removal

**Figure 4:**
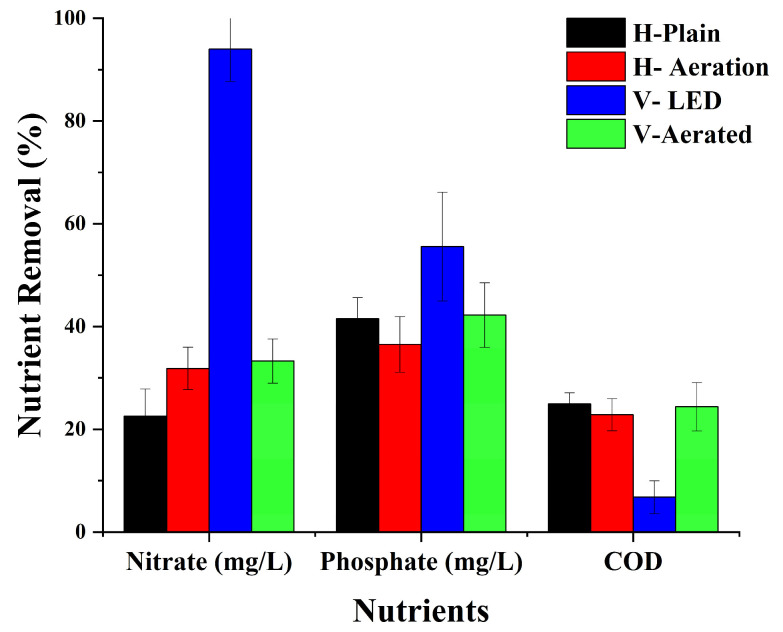
Nutrients removal rate of the four PBR configurations; H-Plain, H-Aerated, V-LED, and V-Aerated.

In addition to biomass production, the cultivation of *Chlorococcum* sp. was evaluated for its ability to remove key nutrients such as nitrate, phosphate, and COD from aquaculture wastewater. Figure 4 illustrates that nutrient removal efficiency varied significantly across different PBR configurations, indicating that reactor design, aeration, and lighting systems affect nutrient uptake and assimilation.

Nitrate removal exhibited the strongest differences among systems, as confirmed by ANOVA (F = 196.89, *p* < 0.001). Tukey’s HSD test showed that V-LED achieved significantly higher nitrate removal (94%) compared to all other systems, while H-Aerated (31.8%) and V-Aerated (33.3%) were statistically similar. H-Plain performed the lowest (22.5%). This outcome highlights that enhanced light quality, rather than aeration alone, was the decisive factor in nitrogen assimilation. Red LED illumination in V-LED likely stimulated nitrate reductase activity and boosted assimilation rates, consistent with prior reports on light-enhanced nitrogen uptake [36,37,38].

Phosphate removal followed a different trend. While V-LED achieved the highest value (55.6%), differences among the systems were not statistically significant (ANOVA: *p* = 0.138). This suggests that phosphate uptake may be less sensitive to PBR configuration and more dependent on internal algal metabolic state. The absence of strong differences also reflects the complex regulation of phosphorus assimilation, which requires additional energy input for transport across membranes [39,40]. Thus, while light quality may provide some advantage, the data show that phosphate removal was not significantly enhanced by red LED supplementation within the experimental window.

COD removal, a measure of organic matter degradation, was also not significantly different among systems (ANOVA: *p* = 0.104). The moderate removals (22–25% in H-Plain, H-Aerated, and V-Aerated; 6.8% in V-LED) likely reflect contributions from both algal and bacterial metabolism. Because the wastewater was non-sterile, bacterial activity was unavoidable. However, the steady increase in DO and algal biomass indicates that *Chlorococcum* sp. dominated system performance, creating competition that limited bacterial proliferation. The relatively lower COD removal in V-LED compared to its strong nitrate uptake suggests that bacterial populations, rather than algae, were more responsible for COD degradation, and their activity may have been suppressed under high-light conditions. However, it is worth noting that it is possible for inhibitory effects to occur at substantially higher COD levels, which should be examined in future work.

Overall, the results demonstrate that V-LED provided the most consistent enhancement of nutrient removal, but only nitrate removal showed statistically significant gains. This reinforces that red LED light strongly influences nitrogen assimilation and phosphorus, while COD removal is governed by additional factors such as algal-bacterial interactions and internal metabolic regulation. Taken together, these results indicate a dual role for red LED illumination in the vertical system: mitigating photolimitation along the column depth and stimulating nitrogen assimilation through enhanced photosynthetic activity. These findings highlight the importance of tailoring PBR design not only for biomass yield but also for nutrient-specific remediation targets.

### 3.4. PBR Geometry Impact

This study evaluated horizontal and vertical PBR configurations to determine how reactor shape, S/V ratio, aeration, and lighting conditions affect the cultivation of *Chlorococcum* sp. Although horizontal and vertical PBRs had similar S/V ratios of 7.69 cm^−1^ and 30.73 cm^−1^, respectively (Table 1), their performance differed significantly. This indicates that S/V ratio alone is not a reliable predictor of cultivation success. Instead, reactor orientation, dimensional characteristics, and operational conditions collectively influence growth outcomes.

The results clearly demonstrate that V-LED, combining vertical reactor geometry with red LED lighting, outperformed all other configurations in terms of biomass concentration (0.50 g L^−1^) and volumetric productivity (0.063 g L^−1^/day), despite its smaller surface area and taller height. This contrasts with H-Plain and H-Aerated, which, despite providing larger surface areas for light absorption and enhanced aeration to improve CO_2_ distribution, did not achieve similar productivity levels.

Horizontal PBRs like H-Plain and H-Aerated offer the advantage of a shallow culture depth (16 cm) and a large illuminated surface area, as the PBRs are effectively laid horizontally and exposed to light from above. In this configuration, the primary light path is from the surface to the bottom across a relatively short distance, which supports good light penetration but still becomes limiting as biomass density increases and self-shading intensifies.

In contrast, for the vertical PBRs, photons must traverse the 90 cm culture height, creating a longer light path and stronger vertical gradients than in the shallow horizontal PBRs. This increases the likelihood of self-shading, particularly in the central and lower regions. The V-LED configuration mitigates these limitations by supplying circumferential red illumination matched to chlorophyll absorption, thereby reducing photolimitation despite the more challenging optical geometry. Internal illumination could further shorten the effective light path but would constitute a different reactor design with separate engineering and operational trade-offs.

Aeration dynamics also showed a clear dependency on reactor geometry. The H-Aerated system, with its wider diameter, achieved peak DO levels of 2.9 mg L^−1^ and a biomass productivity of 0.47 g L^−1^, indicating more effective bubble dispersion and gas retention. In contrast, the vertical reactors, despite continuous aeration at a flow rate of 4 L min^−1^, recorded lower DO concentrations, with a maximum of 1.8 mg L^−1^ in V-LED and even lower in V-Aerated. This suggests that the narrower vertical column results in rapid bubble rise and reduced gas–liquid contact time, limiting oxygen transfer efficiency.

Beyond biological performance, the geometry of the PBRs has practical implications for scale-up and space efficiency in real-world applications. While horizontal systems can provide better light distribution [41,42], their larger footprint may present spatial challenges, especially in indoor cultivation environments. However, the light distribution depends heavily on how the light source is configured and the circulation of the culture media. In contrast, vertical reactors offer a more compact and modular design, making them more scalable, but this comes at the cost of higher energy consumption for lighting and the need for optimized aeration to overcome photolimitation and gas transfer inefficiencies.

For example, while the V-LED system demonstrated the highest productivity, it required approximately 1 kWh of energy (shown in Table 2) for the entire cultivation period, compared to negligible energy input in the H-Plain system, which relies solely on sunlight. This energy cost highlights the trade-off between enhanced productivity and energy consumption in vertical systems. Therefore, maximizing *Chlorococcum* sp. cultivation efficiency requires an integrated design approach that optimizes PBR geometry, lighting strategy, and aeration systems.

The specific aeration rates also differed between configurations, with the horizontal system receiving approximately 0.14 L L^−1^ min^−1^ and the vertical systems receiving 0.29 L L^−1^ min^−1^. These differences likely contributed to the distinct DO patterns and may have influenced cultivation performance alongside geometric and illumination effects.

These findings should therefore be interpreted as indicative trends for two representative sets of operating systems rather than as a strict geometry-only comparison. Because reactor volume, light regime and specific aeration differ between configurations, the individual effects of geometry, aeration and illumination cannot be fully disentangled in the present design. A future factorial study, in which these parameters are varied systematically and independently, will be required to resolve their separate contributions.

### 3.5. Impact of PBR Materials on Chlorococcum sp. Cultivation Efficiency

Material selection influenced light availability, thermal stability, and long-term operability of the PBR systems. Horizontal reactors constructed from stainless steel (SS302) were opaque and reflective, limiting direct light penetration. Illumination entered mainly through the stainless-steel lid, which restricted photon distribution across the broth. Despite this limitation, the shallow culture depth and wide diameter compensated by ensuring a short optical path, allowing sufficient light for photosynthesis. This supported the moderate peak biomass values observed in H-Plain (0.38 g L^−1^) and H-Aerated (0.47 g L^−1^), consistent with Section 3.1. Stainless steel also provided mechanical robustness, resistance to leaching, and reduced fouling potential, making it a reliable option for long-term cultivation and large-volume systems [43,44].

By contrast, vertical reactors were fabricated from transparent acrylic, which maximized light penetration through the full reactor wall. This material advantage was particularly critical given the tall geometry and long optical path of vertical systems. Acrylic facilitated deeper photon delivery, improving the effectiveness of red LED supplementation in the V-LED setup (0.50 g L^−1^). However, acrylic presented operational drawbacks: its smooth surface was prone to biofouling, progressively reducing light transmittance, and its lower thermal conductivity increased the risk of localized heating under continuous illumination [45]. These effects demand regular cleaning and careful monitoring to maintain stable performance.

In summary, stainless steel ensured durability and fouling resistance but required geometric adaptations to counter limited transparency, since typically culture depths are between 10 and 30 cm [46], while acrylic enhanced optical delivery but introduced maintenance challenges. Material choice must therefore balance light transmission with durability and operational stability, especially in scale-up scenarios. Future designs could explore transparent or partially transparent materials for horizontal systems to enhance light penetration and improve comparability with vertical acrylic reactors.

### 3.6. Volumetric Productivity and Electrical Energy Consumption

Biomass productivity, expressed as the daily rate from inoculation to each setup’s peak biomass concentration, was used as a standardized metric to compare photobioreactor (PBR) efficiency across configurations and working volumes and to indicate scaling potential. In this study, yield estimation was computed from the start of cultivation to peak biomass value. Table 2 summarizes the resulting volumetric productivity and daily energy consumption for the four PBR setups. These values represent electrical energy use only; solar input was excluded and would need to be incorporated into a full energy or life-cycle assessment [47].

**Figure 5:**
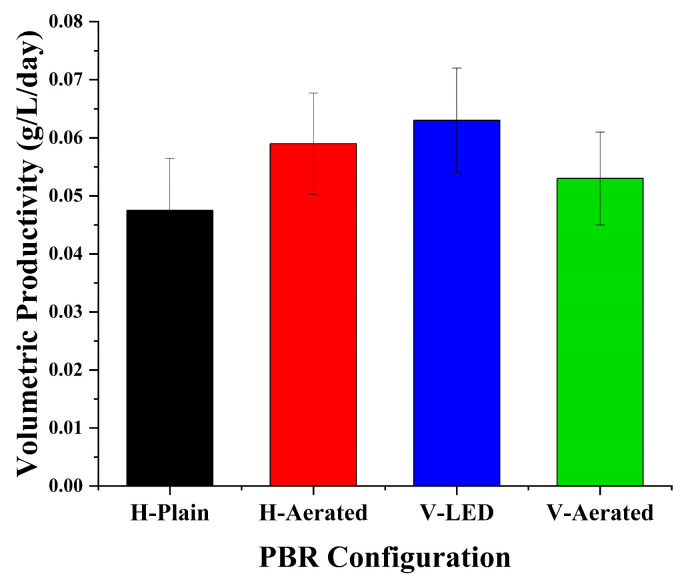
Volumetric productivity.

Volumetric productivity (Table 2; Figure 5) tracked the biomass concentrations (Figure 2), with the ordering V-LED > H-Aerated > V-Aerated > H-Plain. On a day-to-peak basis, the corresponding productivities were 0.063 g L^−1^ day^−1^ (V-LED), 0.059 g L^−1^ day^−1^ (H-Aerated), 0.053 g L^−1^ day^−1^ (V-Aerated), and 0.038 g L^−1^ day^−1^ (H-Plain). The superiority of V-LED is consistent with mitigation of vertical photolimitation via red illumination aligned with chlorophyll absorption, which enhances photon utilization when mixing supplies CO_2_ to the illuminated zone [24,25,26,29]. By contrast, aeration without spectral control (H-Aerated, V-Aerated) increased gas–liquid transfer but yielded lower per-volume gains, in line with reports that mass-transfer improvements translate to biomass only when photons are sufficient and well distributed along the optical path [7,12,33].

As for energy consumption, it diverged sharply across configurations (Table 2). As expected, H-Plain operated at zero external energy (0 kWh day^−1^). H-Aerated consume around 0.24 kWh day^−1^ and V-Aerated drew 0.12 kWh day^−1^ and while V-LED required 1.08 kWh day^−1^ due to the LED load. V-LED also consumed highest energy per volumetric productivity (17.28), followed by H-Aerated (2.043) and V-Aerated (2.286) with H-Plain at 0. Thus, although V-LED offers the strongest per-volume productivity, it does so at the cost of 8 times daily energy consumption of aerated systems. If minimizing power demand is the priority, H-Plain provides zero-energy operation; among powered options, H-Aerated delivers the most favorable energy–biomass balance, whereas V-Aerated incurs half daily energy with slightly lower productivity. Accordingly, configuration choice depends on priority: per-volume productivity favors LED-assisted vertical columns, whereas energy per daily productivity favors larger volume horizontal with aeration system [48,49,50].

**Figure 6:**
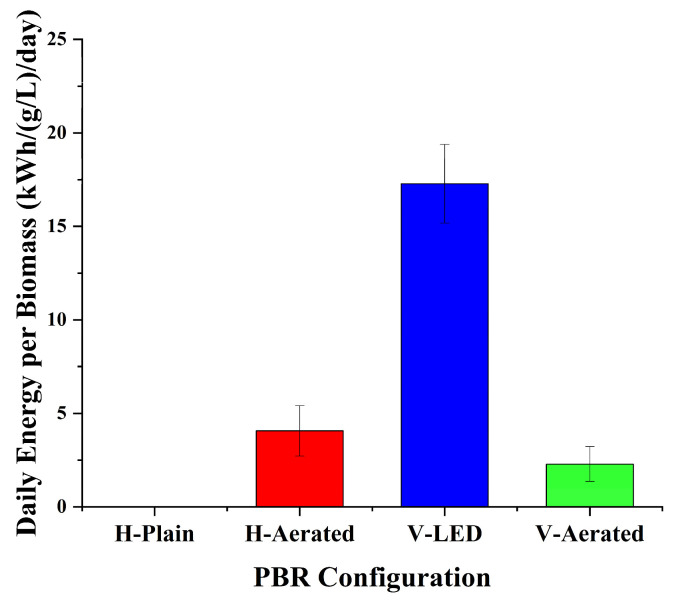
Daily energy consumption per biomass.

Therefore, across configurations, productivity responded more strongly to both aeration and light supply, rather than to aeration intensity alone. Gains from gas transfer required co-optimization with photon delivery to sustain biomass accumulation [7,12,24,25,26,33], as reflected by Table 2 and Figure 5 and in the yield scaling shown in Figure 6.

### 3.7. Implications for Integrated Test Strategy and Scale-Up

The cultivation and nutrient-removal results presented here define quantitative operating windows for biomass productivity, nutrient uptake and specific electrical energy use, providing an initial basis for an integrated test strategy for microalgae-based wastewater treatment, conceptually aligned with the staged testing framework originally proposed for anaerobic wastewater biodegradability assessment [51]. These metrics offer practical guidance on aeration, light delivery and geometry-dependent performance that can inform the design of subsequent pilot-scale trials, where hydraulic loading, influent variability and operational duration are progressively intensified to assess system robustness.

Translation of these findings to real-world applications requires consideration of operational constraints. Horizontal systems align well with existing aquaculture ponds and support low-energy operation where land area is available, while vertical reactors enable higher productivity per footprint at the cost of greater electrical demand. The biomass produced in either configuration can be incorporated into broader resource-recovery schemes within an integrated treatment system.

For upscaling toward full-scale aquaculture or municipal installations, future work should pair these design insights with comprehensive wastewater characterization, long-term pilot operation and environmental and economic assessments to evaluate feasibility under realistic conditions. A further limitation is that the *Chlorococcum* sp. strain used here was identified based on morphology only; sequencing-based characterization will be needed to refine its taxonomic placement and improve comparability across studies. Addressing these aspects will support a robust, stepwise pathway from bench-scale experiments to reliable, scalable treatment units integrated into existing wastewater infrastructure.

## 4. Conclusions

This study confirms that PBR design is a critical factor influencing the cultivation performance of *Chlorococcum* sp. for biomass production and wastewater remediation. Among the four tested configurations, the V-LED system achieved the highest biomass concentration and nutrient removal due to the combined effects of aeration and red LED illumination, whereas the H-Plain system sustained stable growth under natural light with no external energy input. These findings emphasize that reactor geometry, aeration, and light management must be co-optimized to maximize productivity and resource efficiency. Nevertheless, simultaneous variation in design parameters and the use of non-sterile wastewater limited the ability to isolate individual effects, while the higher energy demand of vertical LED systems raises concerns about large-scale feasibility. Accordingly, the present results should be viewed as comparative case studies of practically relevant horizontal and vertical configurations operated under typical local conditions, providing indicative design guidance rather than definitive, parameter-isolated performance limits. These limitations reflect the use of representative operating systems rather than a full factorial design, and future work should employ such controlled experiments to quantify the independent effects of geometry, aeration and illumination. Future work should also incorporate controlled parameter testing, microbial community profiling, more thorough wastewater characterization and life-cycle assessment to evaluate scalability and sustainability.

## Figures and Tables

**Table 1:** PBR geometry and setup; H-Plain, H-Aerated, V-LED, and V-Aerated.

Setup	Surface Area (cm^2^)	Volume (L)	Surface-to-Volume Ratio (cm^−1^)	Aeration (L/min)	Lighting (µmol Photons m^−2^ s^−1^)
H-Plain	4460	58	7.69	None	30
H-Aerated	4460	58	7.69	8	30
V-LED	4241	13.8	30.73	4	53
V-Aerated	4241	13.8	30.73	4	30

**Table 2:** Daily Biomass Productivity in four PBRs; H-Plain, H-Aerated, V-LED, and V-Aerated.

Setup	Peak Biomass Concentration (g L^−1^)	Volumetric Productivity (g L^−1^/day)	Total Electrical Energy (kWh/day)	Daily Electrical Energy per Biomass (kWh/(g L^−1^ day^−1^))
H-Plain	0.38 ± 0.012	0.0475 ± 0.001	0	0
H-Aerated	0.47 ± 0.025	0.059 ± 0.003	0.24	4.067
V-LED	0.5 ± 0.053	0.063 ± 0.007	1.08	17.28
V-Aerated	0.42 ± 0.042	0.053 ± 0.003	0.12	2.286

## Data Availability

The original contributions presented in this study are included in the article. Further inquiries can be directed to the corresponding author.

## Supplementary Materials

The following supporting information can be downloaded at: https://www.mdpi.com/article/10.3390/bioengineering13040388/s1, Table S1: Characterization of wastewater Used for Cultivating *Chlorococcum* sp. Figures S1 and S2: PBR setups. Table S2: One-way ANOVA for biomass concentration across PBR configurations. Table S3: Tukey’s HSD post-hoc test for biomass concentration. Table S4: One-way ANOVA for nutrient removal across PBR configurations. Table S5: Tukey’s HSD post-hoc test for nitrate removal (%). Table S6: One-way ANOVA for OD across PBR configurations. Table S7: Tukey’s HSD post-hoc test for OD. Table S8: Per-configuration models (with intercept).

## Abbreviations

The following abbreviations are used in this manuscript:

ANOVA	Analysis of variance
COD	Chemical oxygen demand
DO	Dissolved oxygen
H-Aerated	Horizontal photobioreactor with aeration
H-Plain	Horizontal photobioreactor without aeration
LED	Light-emitting diode
LEDs	Light-emitting diodes
NO_3_^−^	Nitrate ion
PBR	Photobioreactor
PBRs	Photobioreactors
PO_4_^3−^	Phosphate ion
S/V	Surface-area-to-volume ratio
V-Aerated	Vertical photobioreactor with aeration
V-LED	Vertical photobioreactor with aeration and red LED lighting
kWh	Kilowatt-hour
