# Supplementary material for: Performance Evaluation of Chlorococcum sp. in Various Photobioreactor Designs: Impact on Biomass Production and Nutrient Removal
Source: Bioengineering (Basel). 2026 Mar 27;13(4):388. doi: 10.3390/bioengineering13040388 (PMC13114048; doi:10.3390/bioengineering13040388)
Supplement: Supplementary file 1 [file bioengineering-13-00388-s001.zip › bioengineering-3996243-supplementary.pdf]

Supplementary Data

Table S1: Characterization of wastewater Used for Cultivating *Chlorococcum* sp.

Parameters	H-Plain	H- Aeration	V- LED	V-Aerated
**Nitrate (mg/L)**	63.9	51.5	30	58.6
**Phosphate (mg/L)**	3.76	2.52	1.8	4.12
**COD (mg/L)**	23.4	26.3	59	27.9
**pH**	7.9	7.8	7.9	7.8
**Dissolved Oxygen (mg/L)**	0.9	0.3	1	1
**Temperature (^{0}C)**	22.5	22.5	22.3	22.2

Figure S1 and S2: PBR setups

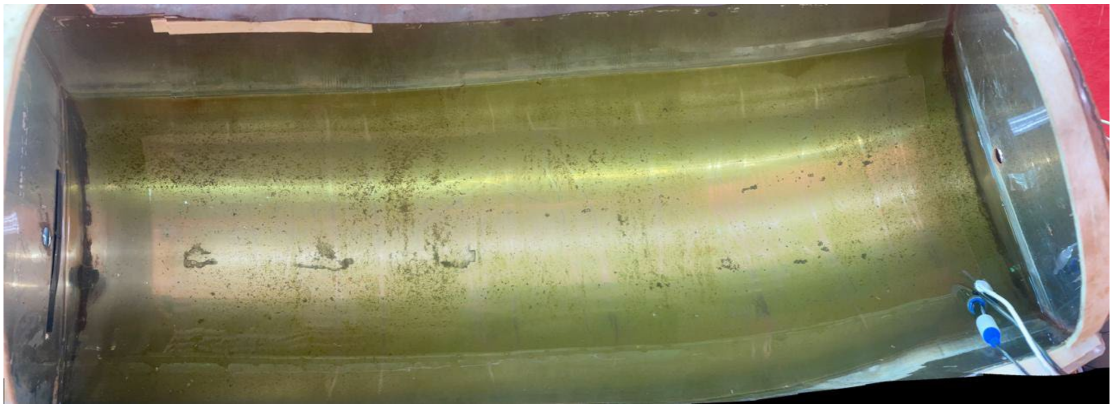

[Figure S1a. H-Plain]

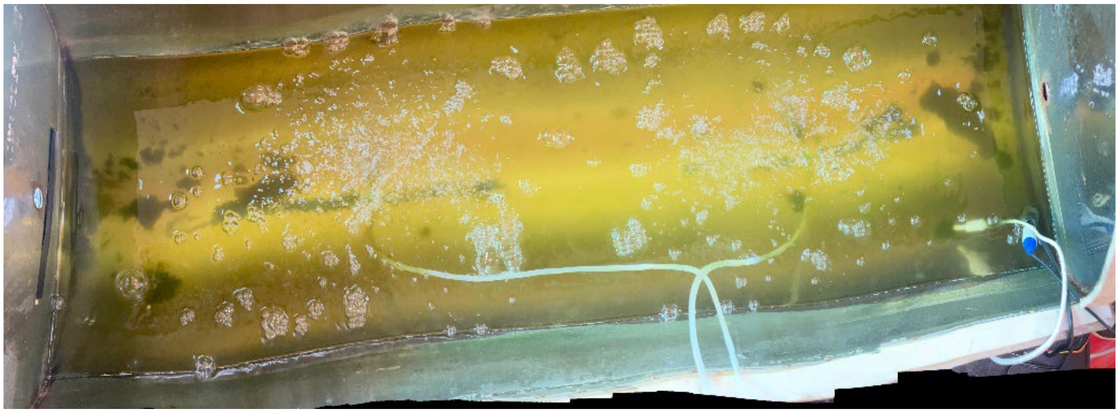

[Figure S1b. H-Aerated]

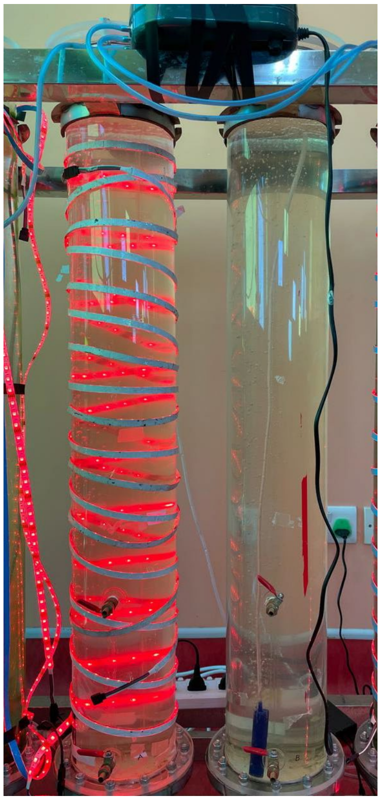

Figure S2. V-LED (left) and V-Aerated (right)

Table S2: One-way ANOVA for biomass concentration across PBR configurations

Source of Variation	df	F	p-value
Between Groups	3	8.651	0.00045
Within Groups	24	–	–
Total	27	–	–

Table S3: Tukey's HSD post-hoc test for biomass concentration

Comparison	Mean Difference	p-adj	95% CI (Lower, Upper)	Significant
**H-Plain vs H-Aerated**	-0.096	0.001	(-0.155, -0.037)	Yes
**H-Plain vs V-LED**	-0.115	0.000	(-0.174, -0.056)	Yes

Comparison	Mean Difference	p-adj	95% CI (Lower, Upper)	Significant
**H-Plain vs V-Aerated**	-0.066	0.034	(-0.125, -0.007)	Yes
**H-Aerated vs V-LED**	-0.019	0.778	(-0.078, 0.040)	No
**H-Aerated vs V-Aerated**	0.030	0.424	(-0.029, 0.089)	No
**V-LED vs V-Aerated**	0.049	0.130	(-0.010, 0.108)	No

Table S4: One-way ANOVA for nutrient removal across PBR configurations

Parameter	F-value	p-value	Interpretation
Nitrate	196.89	7.8×10^{-8}	Significant differences among groups
Phosphate	2.45	0.138	Not significant
COD	2.86	0.104	Not significant

Table S5: Tukey's HSD post-hoc test for nitrate removal (%)

Comparison	Mean Diff	p-adj	95% CI (Lower, Upper)	Significant
**H-Plain vs H-Aerated**	-9.30	0.031	(-17.8, -0.8)	Yes
**H-Plain vs V-LED**	-71.46	0.000	(-79.9, -63.0)	Yes
**H-Plain vs V-Aerated**	-10.74	0.018	(-19.2, -2.3)	Yes
**H-Aerated vs V-LED**	-62.16	0.000	(-70.6, -53.7)	Yes
**H-Aerated vs V-Aerated**	-1.44	0.873	(-9.9, 7.0)	No
**V-LED vs V-Aerated**	60.72	0.000	(52.2, 69.2)	Yes

Table S6: One-way ANOVA for OD across PBR configurations

Source of Variation	df	F-value	p-value
Between Groups	3	22.10	4.36×10^{-7}
Within Groups	24	–	–
Total	27	–	–

Table S7: Tukey's HSD post-hoc test for OD

Comparison	Mean Difference	p-adj	95% CI (Lower, Upper)	Significant
**H-Plain vs H-Aerated**	-0.055	0.004	(-0.094, -0.016)	Yes
**H-Plain vs V-LED**	-0.185	0.000	(-0.224, -0.146)	Yes
**H-Plain vs V-Aerated**	-0.019	0.503	(-0.058, 0.020)	No
**H-Aerated vs V-LED**	-0.130	0.000	(-0.169, -0.091)	Yes
**H-Aerated vs V-Aerated**	0.036	0.149	(-0.003, 0.075)	No
**V-LED vs V-Aerated**	0.166	0.000	(0.127, 0.205)	Yes

Table S8: Per-configuration models (with intercept)

Configuration	Slope	Intercept	R^2	RMSE (g/L)
**H-Plain**	2.879	0.042	0.811	0.040
**H-Aerated**	1.205	0.249	0.501	0.038
**V-LED**	0.355	0.324	0.519	0.032
**V-Aerated**	1.166	0.269	0.443	0.040

H-Plain: Biomass = 2.879×OD + 0.042 (R^2 = 0.811; RMSE = 0.040 g L^{-1})
H-Aerated: Biomass = 1.205×OD + 0.249 (R^2 = 0.501; RMSE = 0.038 g L^{-1})
V-LED: Biomass = 0.355×OD + 0.324 (R^2 = 0.519; RMSE = 0.032 g L^{-1})
V-Aerated: Biomass = 1.166×OD + 0.269 (R^2 = 0.443; RMSE = 0.040 g L^{-1}).
